# Muscle-inspired soft robots based on bilateral dielectric elastomer actuators

**DOI:** 10.1038/s41378-023-00592-2

**Published:** 2023-10-07

**Authors:** Yale Yang, Dengfeng Li, Yanhua Sun, Mengge Wu, Jingyou Su, Ying Li, Xinge Yu, Lu Li, Junsheng Yu

**Affiliations:** 1 State Key Laboratory of Electronic Thin Films and Integrated Devices, School of Optoelectronic Science and Engineering, University of Electronic Science and Technology of China (UESTC), Chengdu, PR China; 2 Chongqing Key Laboratory of Materials Surface & Interface Science, Chongqing Co-Innovation Center for Micro/Nano Optoelectronic Materials and Devices, Micro/Nano Optoelectronic Materials and Devices International Science and Technology Cooperation Base of China, School of Materials Science and Engineering, Chongqing University of Arts and Sciences, Chongqing, PR China; 3 Hong Kong Centre for Cerebro-Cardiovascular Health Engineering (COCHE), Hong Kong, SAR China; 4 Department of Biomedical Engineering, City University of Hong Kong, Hong Kong, SAR China

**Keywords:** Electronic properties and materials, Electrical and electronic engineering

## Abstract

Muscle groups perform their functions in the human body via bilateral muscle actuation, which brings bionic inspiration to artificial robot design. Building soft robotic systems with artificial muscles and multiple control dimensions could be an effective means to develop highly controllable soft robots. Here, we report a bilateral actuator with a bilateral deformation function similar to that of a muscle group that can be used for soft robots. To construct this bilateral actuator, a low-cost VHB 4910 dielectric elastomer was selected as the artificial muscle, and polymer films manufactured with specific shapes served as the actuator frame. By end-to-end connecting these bilateral actuators, a gear-shaped 3D soft robot with diverse motion capabilities could be developed, benefiting from adjustable actuation combinations. Lying on the ground with all feet on the ground, a crawling soft robot with dexterous movement along multiple directions was realized. Moreover, the directional steering was instantaneous and efficient. With two feet standing on the ground, it also acted as a rolling soft robot that can achieve bidirectional rolling motion and climbing motion on a 2° slope. Finally, inspired by the orbicularis oris muscle in the mouth, a mouthlike soft robot that could bite and grab objects 5.3 times of its body weight was demonstrated. The bidirectional function of a single actuator and the various combination modes among multiple actuators together allow the soft robots to exhibit diverse functionalities and flexibility, which provides a very valuable reference for the design of highly controllable soft robots.

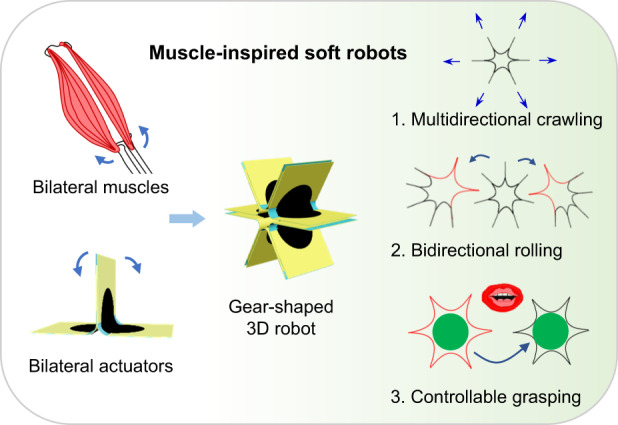

## Introduction

The emergence of developing soft robots provides a successful example of learning from nature to replicate human or animal behaviors and functions^1^. Conventional robots designed with rigid bodies and mechanical actuation units often exhibit precise control and movement, but there is still a gap between them and real animals or humans stemming from the difference in the bionic structure^2^. The human body structure contains not only hard bones as the support frame of the body but also soft muscles and joints as the soft actuators and controllers for body movements. Designing soft actuators to mimic the animal body construction and kinematics^3–5^ is an effective complement to robotic bionics. Soft actuation technology has been developed with various intelligent materials, such as dielectric elastomers (DEs)^6,7^, liquid crystalline polymers (LCPs)^8,9^, shape memory alloys (SMAs)^10^, and hydrogels^11^. Soft actuators and robots are playing an irreplaceable role in biomedical engineering^12,13^, marine detection^14,15^, medical rehabilitation^16^, and industrial operations^7,17^ due to the softness of their bodies. To present good maneuverability and motion performance, the controllability of the soft robot is an extremely important indicator.

Robots often perform precise multidimensional control by relying on accurate motors or mechanical devices, while novel types of soft robots can only be controlled by material deformation of soft actuators with a simple actuation method^18,19^. However, the nonlinear deformation of soft materials is difficult to predict^20^, which makes it difficult for soft robots to deal with complex operating requirements^21,22^. To improve the controllability of soft robots, we need to learn from nature to design soft actuators, such as imitating the structure and function of muscles. The muscles of organisms are usually controlled in the form of muscle pairs or muscle groups. The muscles on both sides of a bone have an actuation effect and act in a synergistic manner when limbs move. Taking the human body as an example, bilateral muscles include the biceps brachii and triceps brachii, interosseous muscles, orbicularis oculi muscles, etc. The interosseous muscles can control the swinging of fingers, and the orbicularis oculi muscles can cooperate to control the opening and closing of eyes^23,24^. Therefore, inspired by the structure of muscle groups, increasing the control dimension is an effective strategy to design controllable soft actuators and robots.

Here, we report a highly controllable soft robot based on a bilateral actuation structure. Similar to the muscle pair of organisms, the bilateral actuators present a two-way control dimension and coordinated behavior similar to human musculature. Each bilateral actuator is assembled from two unilateral actuators made of low-cost VHB 4910 dielectric elastomer and polyethylene terephthalate (PET). A dielectric elastomer^25^ is regarded as an ideal choice to fabricate artificial muscle. Compared with photothermal actuators^26^ and humidity-responsive actuators^27^, dielectric elastomer actuators have advantages such as a fast response^28^ and electrical controllability. Compared to other electric-powered actuators^29^, dielectric elastomers have the advantage of large deformation^28^. Furthermore, we constructed a gear-shaped 3D robot employing these bilateral actuators to demonstrate more versatile functions and controllability. As moving robots, the assembled 3D soft robots exhibit good controllability in crawling and rolling. The crawling robot has a multidirectional movement ability through direct switching among the bilateral actuators. Due to the characteristics of the bilateral actuation, the rolling robot can achieve bidirectional rolling motion and climbing motion on a 2° slope. Finally, a mouthlike soft robot was designed as a gripper to bite and grasp objects five times its own weight. Many existing soft robots also have forms of motion such as crawling^30,31^ and rolling^32^. Most of them have only one motion mode, while our gear-shaped 3D robots have multiple functions and higher controllability and maneuverability (Table S1). Inspired by muscle groups, the design of this bilateral actuator provides a novel idea for developing highly controllable 3D robots.

## Results and discussion

### Bilateral soft actuators

**Fig. 1:**
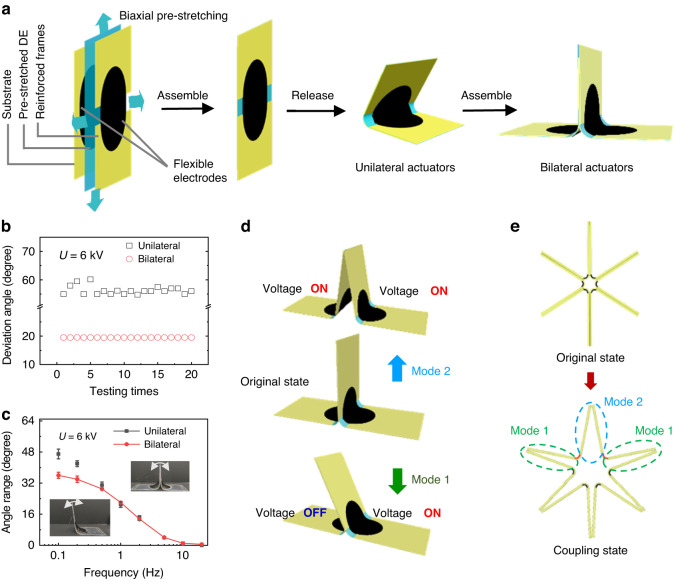
Design and fabrication of the muscle-inspired bilateral actuator. **a** Fabrication process of the bilateral actuator. **b** Stability performance test of the unilateral actuator and the bilateral actuator. **c** Changes in the deviation angle range with the actuation frequency. **d** Two actuation modes for the bilateral actuator. Mode 1 refers to the actuation mode of applying a voltage on either side to generate unilateral bending. Mode 2 refers to the actuation mode of applying the same voltage on both sides to generate a gap. **e** Design of the gear-shaped 3D robot with the coupling of mode 1 and mode 2

Figure 1a and Supplementary Fig. S1 show the manufacturing process of unilateral actuators and bilateral actuators. The unilateral actuator consists of a 0.18 mm-thick PET substrate, 0.18 mm-thick reinforcement frames (Fig. S3), and a prestretched dielectric elastomer with double-sided carbon grease electrodes as an active layer. After release, the dielectric elastomer forms a saddle surface, and the actuator is in a curved shape with a certain angle. When a voltage is applied to the carbon grease electrodes on both sides, the DE film subjected to Maxwell stress shrinks in the direction of the electric field and expands in the direction perpendicular to the electric field. At the same time, the elastic potential energy stored in the PET frame is released, and the actuator tends to flatten. The bilateral actuator was formed by bonding two unilateral actuators in a back-to-back manner (Fig. 1a).

Compared to the unilateral actuator, the bilateral actuator presents better performance in stability and dimensional control. First, the bilateral actuator can still work as a unilateral actuator by only actuating one side of the actuator. The deviation angle, defined as the angle change of the actuators relative to their original state, was introduced to evaluate the performance of the actuator. When the actuator bends to the right side, the value of the deviation angle is set to be positive, and it was set to negative for left-side bending. Through experiments, we found that a working voltage above 6 kV will cause wrinkling or breakdown of the dielectric elastomer, which is unfavorable for the actuator. Therefore, we chose actuating voltages below 6 kV. To test the stability of the actuators, a 6-kV voltage was applied on the unilateral actuator and on the left side of the bilateral actuator for a cycling test with 20 cycles. As shown in Fig. 1b, the bilateral actuator shows better stability, with the standard deviation of the deviation angle being only 0.021°, which is much lower than that of the unilateral actuator with a standard deviation of 1.48°. The stability of the bilateral actuator is attributed to the joint action of the structures on the two sides. However, due to the binding effect of the bilateral structure, the structure on one side of the bilateral actuator will block the deformation of the structure on the other side. Under the same actuation voltage on one side, the bilateral actuator obtains a smaller deviation angle of 19.50° than that of 56.31° for the unilateral actuator. The bilateral actuator can also work as a bilateral bending actuator by actuating both sides of the actuator in an alternating manner, exhibiting enhanced dimensional control. In contrast, the unilateral actuator can only bend toward one side. The angle range was introduced to define the range of the deviation angle of the actuator. As shown in Fig. 1c and Movie S1, both the unilateral and bilateral actuators demonstrate a decrease in the angle range with increasing actuation frequency. The bilateral actuator can still generate deviation angles at higher frequencies ( > 10 Hz), which makes it possible to use it in underwater swimming robots. Many studies have shown that as long as the encapsulation is optimized, underwater robots are possible^14,33^. This deformation law with the actuation frequency provides an important data reference for the design and actuation of subsequent robots.

As described above, the bilateral actuator can deform in two directions by alternately actuating the two sides of the actuator, which can be defined as mode 1. When both sides of the actuator are simultaneously actuated, the middle of the actuator splits and creates a certain gap, which can be defined as mode 2 (Fig. 1d). The size of the gap is called the gap distance. By integrating the bilateral actuators, a 3D gear-shaped robot can be created (Fig. 1e). Actuation mode 1 and mode 2 can be combined to adjust the shape and posture of the robot to realize highly controllable multidirectional movement.

The performance of the bilateral actuator is related to the stiffness of the PET and the prestretching ratio of the elastomer. The thickness and hole radius R of the PET substrate are the main factors affecting its stiffness. We use the variation range of the deviation angle (angle range) and the variation range of the gap distance (gap range) to evaluate the impact of these parameters on the performance of the bilateral actuator (Fig. S2a). As shown in Fig. S2b, a larger radius corresponding to a lower stiffness results in a larger angle range and a smaller gap range. For thicker substrates, a larger radius is required to achieve the same performance. In addition, a larger prestretching ratio of the elastomer generates greater deformation of the actuator (Fig. S2c). Therefore, we chose a hole radius of 12.5 mm for the 0.1 mm-thick PET substrate and 15 mm for the 0.18 mm-thick PET substrate and a 400 × 400% prestretching ratio of the dielectric elastomer for further research.

To better understand the two actuation modes of the bilateral actuator, we conducted finite element analysis for the actuator under the two actuation modes by using ABAQUS software (Fig. S5). The simulation results show that the voltage on one side in mode 1 leads to an asymmetric distribution of stress and strain in the actuator, which results in a deviation angle. In mode 2, when both sides are simultaneously actuated, the stress caused in the middle of the actuator results in a gap distance.

**Fig. 2:**
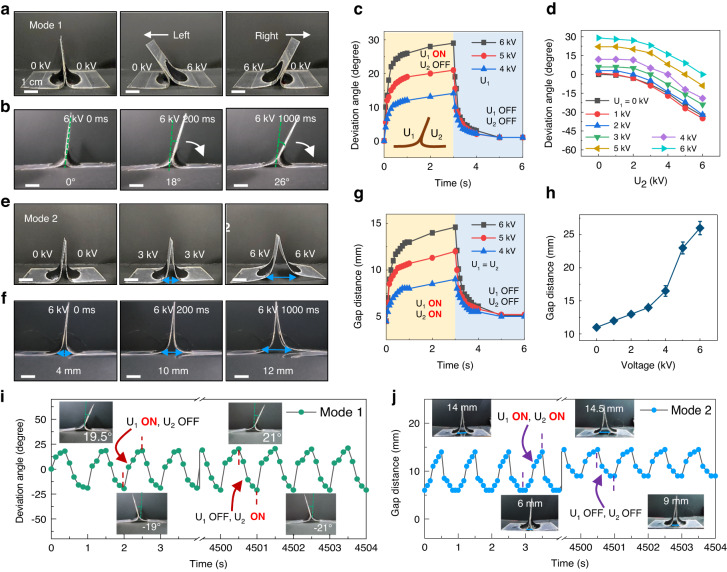
Performance of the bilateral actuator. **a** Bilateral deformation of the bilateral actuator under actuation mode 1. **b** Optical images of the bilateral actuator deformation at 0 ms, 200 ms, and 1000 ms (mode 1). **c** Deviation angle changing with time under actuation mode 1 with left-side voltages *U*_1_ of 4 kV, 5 kV, and 6 kV. **d** Deviation angle of the bilateral actuator under different combinations of *U*_1_ and *U*_2_. **e** Deformation of the bilateral actuator under actuation mode 2. A greater voltage results in a larger gap distance. **f** Optical images of the deformation of the bilateral actuator at 0 ms, 200 ms, and 1000 ms (mode 2). **g** Gap distance changing with time under actuation mode 2 with actuation voltages of 4 kV, 5 kV and 6 kV. **h** Gap distance of the bilateral actuator under different voltages. **i** Cycling performance test of the bilateral deformation in mode 1. The bilateral actuator was actuated by left and right alternating voltages with a value of 5.5 kV and a frequency of 1 Hz. **j** Cycling performance test of the bilateral actuator in mode 2 with actuation voltages of 5.5 kV and a cycling frequency of 1 Hz

Figure 2 summarizes the deformation performance of the bilateral actuator under the two different actuation modes. As shown in Fig. 2a, the bilateral actuator bends to the right when its left side is actuated, and it bends to the left when its right side is actuated. Under an actuation voltage of 6 kV, the deviation angle reaches 18° and 26° at actuation times of 0.2 s and 1 s, respectively (Fig. 2b). The relationship between the deviation angle and actuation time during the bending process is shown in Fig. 2c. The actuator reaches its maximum deformation at approximately 1.5 s. The actuation voltage on the left side is expressed as *U*_1,_ and that on the right side is expressed as *U*_2_. This alternating high-voltage actuation on either side corresponds to actuation mode 1. Any desired bending angle between −30° and 30° can be acquired by combining different values of *U*_1_ and *U*_2_ (Fig. 2d). When both sides are simultaneously actuated (actuation mode 2), the middle of the bilateral actuator starts to splay, with the formation of a gap, because the actuator is bound to a flat surface (Fig. 2e). The original gap distance is 4 mm and then reaches 10 mm and 12 mm at actuation times of 0.2 s and 1 s, respectively (Fig. 2f). The change in the gap distance with time and with the actuation voltage is shown in Fig. 2g, h. Figure 2i, j demonstrates the cycling performance test of the bilateral actuator under the two actuation modes with an actuation voltage of 5.5 kV and a frequency of 1 Hz. After 4500 cycles of actuation, the deviation angle of the bilateral actuator in actuation mode 1 does not show a decrease but a slight increase from 19° to 21°. Under actuation mode 2 for 4500 cycles, the gap distance of the actuator also increases from 14 mm to 14.5 mm (Movie S2). In addition, as a basic mechanical parameter, we measured the actuation force of the bilateral actuator in the different modes. As shown in Fig. S4, the actuation force generated by the actuator is at the millinewton level. As the voltage increases, the blocking force of the actuator continues to increase. The actuator working in mode 1 produces less actuation force than that working in mode 2 because mode 2 has voltage actuation on both sides. This result ensures that the bilateral actuator can be used for quite long cycles without any degradation in the deformation performance.

### Crawling soft robots

**Fig. 3:**
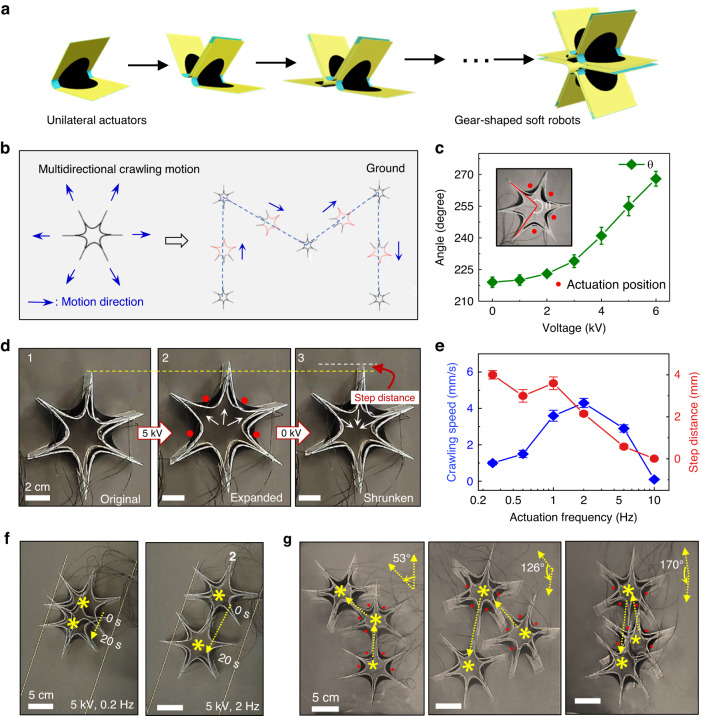
Gear-shaped 3D soft robot and its crawling behavior. **a** Fabrication of the gear-shaped 3D soft robot. **b** Display of the multidirectional controllability and crawling motion along a specified path. **c** Changes in the angle between two bilateral actuators (marked by the red line) with the actuation voltage from 0 kV to 6 kV. **d** Crawling process of the gear-shaped soft robot during one step. The red dots represent the positions of the actuators being actuated. **e** Relationships of the crawling speed and step distance of the gear-shaped soft robot with the frequency at which the actuation voltage is turned on and off. **f** Demonstration of the crawling distance of the robot at different frequencies. **g** Directional controllability of the crawling robot. The crawling orientation of the robot was changed by switching the actuation positions

Based on the deformation performance of the bilateral actuator under the two actuation modes, the coordinated operation of multiple actuators is expected to achieve a robot with high controllability. Figure 3a demonstrates a 3D gear-shaped soft robot integrated with multiple actuators by connecting them end to end to form a closed-ring robot. As shown in Fig. 3b, this soft robot is lying on the ground with all the actuator feet on the ground. When the actuators on the soft robot are actuated, the robot feet can generate a continuous swinging motion, similar to the crawling of some reptiles, such as sea turtles^34^. Figure 3b demonstrates a schematic diagram of the multidirectional crawling motion of the gear-shaped 3D soft robot. By adjusting and switching the positions of the actuated actuators, the soft robot can realize motion in six directions. Thus, the soft robot can crawl along a specified path. By actuating four adjacent actuators, the robot body will expand, which is reflected in the change in the angle between the robot feet (Fig. 3c). The red dots represent the positions of the actuators being actuated. The greater the actuation voltage is, the greater the opening degree and angle changes of the robot.

Figure 3d shows the crawling process of the gear-shaped soft robot during one step. Directional crawling originates from the difference in the direction and duration of the frictional force during the application and removal of a voltage, and we provide a detailed discussion in Fig. S6. At the beginning, the four actuators marked by red dots were actuated with a voltage of 5 kV, and the robot body began to expand and move forward with a significant displacement. After removal of the applied voltage, the robot body began to shrink within a short time and moved backward with a small displacement. The step distance of the robot corresponds to the difference between the forward displacement and the backward displacement. The uneven friction during the forward and backward processes results in a forward step distance. The crawling speed and step distance of the robot are closely related to the frequency at which the actuation voltage is turned on and off (Movie S3). Figure 3e shows the relationships of the crawling speed and step distance of the gear-shaped soft robot with this frequency. The results show that the robot reaches the maximum speed at a frequency of 2 Hz, and the crawling speed is 4.3 mm/s. The step distance of the robot gradually decreases as the switching frequency increases due to the shortening of the deformation time. The step distance of the soft robot reaches a reasonable value of 6 mm. Thus, even under a low actuation frequency of 2 Hz, the crawling speed of this robot still reaches 4.3 mm/s, which is sufficient to generate continuous directional motion (Table S1). As shown in Fig. 3f, this soft robot crawled forward within 20 s under two different frequencies, with a longer moving distance achieved at 2 Hz.

Importantly, the gear-shaped robot has multidirectional symmetry. Therefore, we can actuate the actuators at different positions to make the robot move in different directions (Movie S4 and Fig. 3g). We demonstrated steering control of the robot with several different moving steering angles of 53°, 126°, and 170°, which are close to 60°, 120°, and 180°. The red dots indicate the positions of the actuated actuators for moving in different directions. This switching of the direction of motion is done instantaneously, which shows that the direction switching speed can be very fast. Moreover, the robot can immediately move forward in the next set direction without a circular arc transition. Therefore, in terms of the steering response speed and steering efficiency, this robot is superior to previous robots that rely on body bending for rotation (Table S1)^35–38^.

### Rolling soft robot

**Fig. 4:**
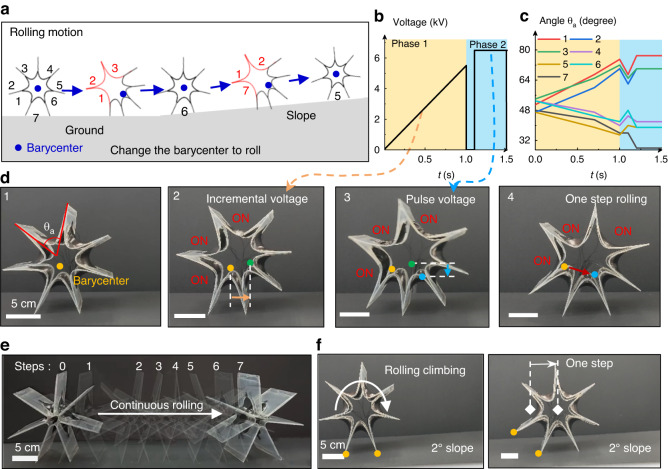
Rolling soft robot. **a** Schematic diagram of the rolling motion of the rolling soft robot on flat ground and a slope. The actuators on the 3D rolling soft robot are numbered clockwise from 1 to 7. **b** Voltage control process for the rolling robot during an actuation period. After the barycenter of the robot rises in phase 1, a pulse voltage in phase 2 makes the robot roll forward. **c** Angle change of each actuator during an actuation period. **d** Motion state analysis of the rolling robot during an actuation period, in which 1 is the start state, 2 is the barycenter rising state, 3 is the rolling state excited by a pulse voltage, and 4 is the end state. **e** Continuous rolling process of the soft robot. **f** Rolling behavior of the soft robot on a 2° slope

The gear-shaped soft robot can also operate in a standing posture with only two feet on the ground. In this way, it can roll forward as a rolling soft robot. By actuating the actuators at different positions, the barycenter of the whole robot can be adjusted to generate a rolling behavior on the ground or even a slope (Fig. 4a). Seven actuators were assembled to produce this rolling soft robot (Supplementary Fig. S8a). To provide guidance for the rolling process, we further investigated the distribution of the soft robot barycenter under different combinations of actuated actuators (Fig. S7 and Fig. S8b). As shown in Supplementary Fig. S8b, when actuators 1, 2, and 3 were actuated, the robot barycenter was lower than that of its original state, and there was also a large displacement along the lateral direction, which provided an important basis for the robot to roll forward. In detail, the rolling process of the robot for one step was recorded and analyzed as follows. The actuation voltage control and angle of all actuators are shown in Fig. 4b, c. The angle of the actuator is the angle between the two legs of the robot where the actuator is located. One step was divided into two phases, called the prerolling phase (phase 1) and pulse phase (phase 2). In the prerolling phase, a voltage that gradually increased from 0 to 5.5 kV was applied to actuators 1, 2 and 3, and the barycenter of the robot moved a large step forward (Fig. 4d). In the pulse phase, a pulse voltage with a peak value of 6.5 kV was applied to generate a momentary excitation to make the robot barycenter rapidly move forward and downward, which caused the robot to roll and take a step forward (Fig. 4d). Similarly, when actuators 4, 5 and 6 were actuated, the soft robot could roll backward (Movie S5). As shown in Table S1, the bidirectional rolling behavior of this rolling soft robot significantly enhanced the motion controllability compared to the unidirectional rolling motion of previous DE-based soft robots.

After the first step, actuators 7, 1, and 2 can be actuated, and the robot will realize a second step forward (Fig. 4a). By repeating this process, the robot can continuously roll forward or backward. Figure 4e and Supplementary Movie S6 show the controlled rolling motion of the soft robot for a complete circle with 7 steps. To characterize the rolling ability of the rolling robot on a slope, we placed the rolling robot on a slope with an inclination angle of 2° for the rolling test (Fig. 4f and Movie S7). The rolling soft robot also exhibited stable rolling motion on slopes, which significantly expands its applicable scenarios.

### Mouthlike soft robot

**Fig. 5:**
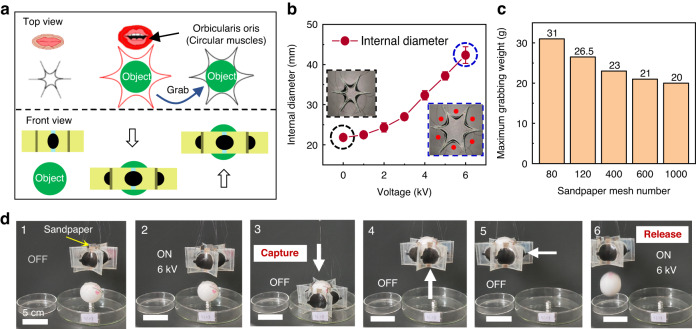
Mouthlike soft robot. **a** Schematic diagram of the grabbing behavior based on the mouthlike soft robot. Imitating the orbicularis oris muscle of the mouth, this robot can open its body, loop around an object, and contract its body to finally grab the object. **b** Changes in the internal diameter of the mouthlike robot with the actuation voltage. The left insert image shows the original state of the mouthlike robot. The right insert image shows the robot actuated by a voltage of 6 kV. **c** Maximum grabbing weight of the mouthlike robot with different sandpapers as the inner wall. **d** Transportation process of a 31 g, 40 mm-diameter table tennis ball by the mouthlike robot

This gear-shaped 3D soft robot exhibits body expansion and contraction during its actuation process, which is ideal for grasping objects. Some researchers developed robots for grasping tasks by imitating human fingers^39,40^. In fact, in addition to fingers, many people also use their mouths to pick up objects, especially disabled persons who have lost their hands. The mouth grasps objects by using the cooperation of circular muscles composed of multiple muscles. In the mouth, the circular muscles are called the orbicularis oris muscle^41^, which is usually controlled to grasp food. The gear-shaped robot is an annular structure connected by multiple actuators. The structure formed by connecting the head and tail of bilateral actuators in series can be regarded as an “artificial circular muscle”. By actuating all actuators, the soft robot can achieve similar grasping functions as a human mouth and can be called a “mouthlike soft robot” (Fig. 5a).

This mouthlike soft robot can adjust its internal size by controlling the actuated voltages on all actuators. The internal diameter was introduced to evaluate the internal size of the mouthlike soft robot, which was defined as the diameter of the largest circle that can be accommodated internally. As shown in Fig. 5b, the robot considerably expanded its body, with the internal diameter doubling from 21 mm to 42 mm, when the actuated voltage increased from 0 kV to 6 kV. To realize effective grabbing performance, an inner wall needs to be added to the actuator, which could provide an insulating contact surface between the robot and the object to protect the actuator electrodes from damage. Here, sandpaper with different mesh numbers was used as the inner wall, and a 40 mm-diameter table tennis ball was used as the grabbed object. During the grabbing performance test of the mouthlike soft robot, the weight of the object was adjusted by injecting water into the table tennis ball. As shown in Fig. 5c, the smaller the sandpaper mesh number is, the heavier the objects that can be picked up. When 80 mesh sandpaper was used, the mouthlike soft robot could grasp a mass of 31 g (5.3 times the mass of the robot). Figure 5d and Supplementary Movie S8 demonstrate the transportation process of a 31 g table tennis ball by this mouthlike soft robot. First, 6 kV voltages were applied to all actuators, and the internal diameter of the robot increased to more than 40 mm. Then, the robot was lowered down so that the inner wall of the robot could wrap the table tennis ball. After the voltages were removed, the inner wall closely stuck to the table tennis ball. At this time, the robot was lifted up, with the friction between the inner wall and the surface of the table tennis ball being sufficient to overcome the gravity of the table tennis ball. After the ball was grabbed, it was transferred and released into another petri dish by applying 6-kV voltages again to open the mouthlike soft robot.

Overall, this gear-shaped 3D soft robot has demonstrated a variety of soft robot motion forms and a high degree of controllability. However, to expand its application range and scenarios, there is still much work that needs to be done to compensate for the shortcomings of this robot. For example, the current robot still requires actuation from external bulky power equipment, which limits its outdoor applications. Moreover, the current motion mode switching still relies on manual control of the power source, and it is not possible to automatically switch between different motion modes. Therefore, in further study, batteries and control circuits can be added to achieve untethered actuation, remote control, and automatic mode switching.

## Conclusion

In this work, we developed a muscle-inspired soft bilateral actuator composed of two DE-based unilateral actuators. The bilateral actuator is superior to the unilateral actuator in stability and dimensional control. For the bilateral actuator, the deformation performance under two actuation modes was studied. To better demonstrate the high controllability of bilateral actuators, we designed a 3D gear-shaped soft robot integrated with bilateral actuators. The gear-shaped soft robot demonstrated outstanding flexibility and controllability in locomotion and object manipulation. As a moving robot, it performed a multidirectional crawling motion with a crawling speed of 4.3 mm/s and excellent steering performance. As a rolling soft robot, it also produced a stable bidirectional rolling motion and stable rolling on a 2° slope. As a mouthlike robot, it could grab and transport a table tennis ball 5.3 times its own weight. Based on the bilateral actuators, the 3D soft robot shows good maneuverability and high controllability in various control scenarios, especially in multidirectional crawling, fast turning, and bidirectional rolling. In summary, our robot provides an important design for highly controllable bionic 3D soft robots.

## Materials and methods

### Fabrication of the bilateral soft actuators and gear-shaped soft robots

First, a 0.18 mm or 0.1 mm thick PET film was cut into specific shapes using a laser cutting machine (Mintron MC-3020). The specific parameters designed with CAD software can be found in Fig. S3. The cut PET films were divided into two shapes: flexible substrates with an elliptical hole and reinforced frames with a semicircular hole. A VHB4910 elastomer (3 M 60 mm × 60 mm) was stretched to 400 × 400% using a prestretching tool. Then, the film was fixed with an acrylic frame and removed from the prestretching tool. Next, a PET substrate and two reinforced frames with flexible wires were adhered to the center of a DE film, and their holes were aligned during adhesion. A thin carbon grease (AMKE G-660A) electrode layer was applied on both sides of the DE film in the hole area with a soft bristle brush, and then, the actuator was removed from the DE film. The unilateral soft actuator was thus acquired. Finally, two unilateral actuators were bonded in a back-to-back manner to obtain the bilateral actuator. The gear-shaped soft robot was obtained by integrating several actuators into a ring structure in a similar way.

### Actuation and test method

We used MOS relays, dry-reed relays, and a microcontroller unit (MCU) to build a simple multichannel high-voltage control circuit (Supplementary Fig. S9) to control the charging and discharging of each actuator. The control of one actuator requires two-channel voltage control, one for charging and the other for discharging. The MCU can control the duty cycle and frequency of each channel voltage through programming.

For mode 1 of bilateral actuators, the two actuators had a common negative pole, the positive pole was connected to a four-channel high-voltage control system, whereas mode 2 required only two-channel high-voltage control. The actuation of each actuator of the crawling robot, rolling robot and mouthlike robot was synchronous, so only two-channel high-voltage control was needed. The frequency in the frequency test of bilateral actuators, the frequency in the cycling test and the actuating frequency of the crawling robot were controlled by MCU. In the test of the mouthlike robot, the weight of the table tennis ball was controlled by injecting water. All videos and images were taken by a camera (OPPO).

### Supplementary information

Supplementary Information

Movie S1

Movie S2

Movie S3

Movie S4

Movie S5

Movie S6

Movie S7

Movie S8
