# Supplementary material for: Muscle-inspired soft robots based on bilateral dielectric elastomer actuators
Source: Microsyst Nanoeng. 2023 Oct 7;9:124. doi: 10.1038/s41378-023-00592-2 (PMC10560252; doi:10.1038/s41378-023-00592-2)
Supplement: Supplementary file 1 — Supplementary Information [file 41378_2023_592_MOESM1_ESM.docx]

**Supplementary Information for**

**Muscle-inspired soft robots based on bilateral dielectric elastomer actuators**

Yale Yang^1,2^, Dengfeng Li^3*^, Yanhua Sun^1,2^, Mengge Wu^1,4^, Jingyou Su^4^, Ying Li^2^, Xinge Yu^4*^, Lu Li^2,*^, Junsheng Yu^1,*^

* Corresponding author. Email: [dengfli2-c@my.cityu.edu.hk](mailto:dengfli2-c@my.cityu.edu.hk) (D.L.), [xingeyu@cityu.edu.hk](mailto:xingeyu@cityu.edu.hk) (X.Y.), [lli@cqwu.edu.cn](mailto:lli@cqwu.edu.cn) (L.L.), [jsyu@uestc.edu.cn](mailto:jsyu@uestc.edu.cn) (J.Y.)

**1. Supplementary Figures Description**


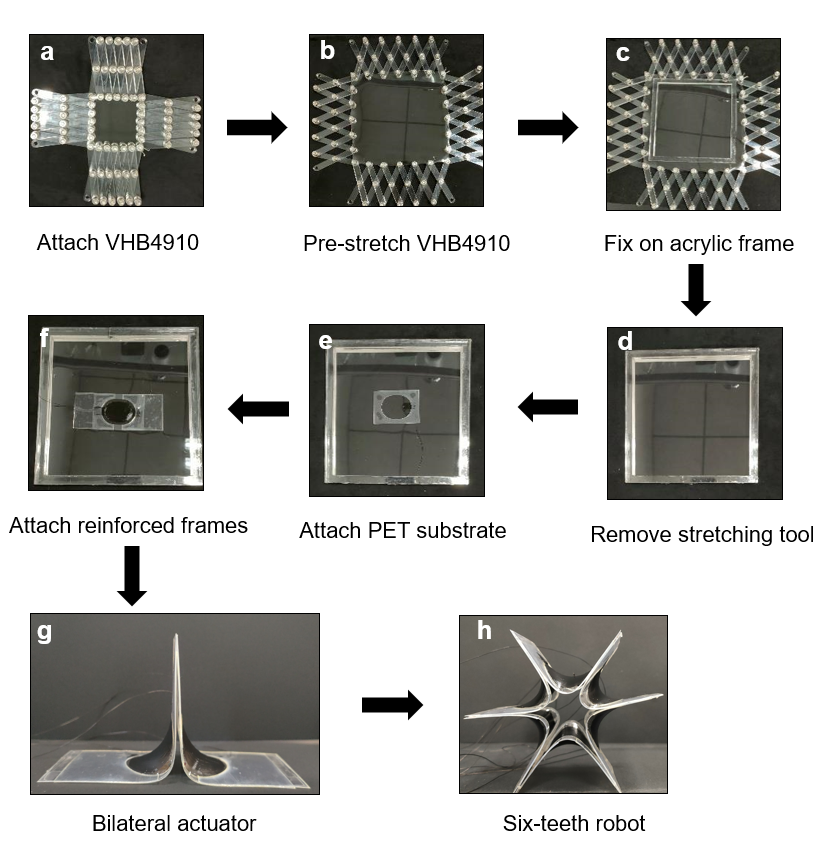


**Fig. S1.** This figure shows the physical flow chart of the fabrication process for the highly controllable soft robots. a) First, paste VHB4910 on a self-made pre-stretching device. b) Use the pre -stretching device to stretch the VHB film to 400%×400%. c) Acrylic frame is used to fix the deformation of the VHB film. d) Remove the acrylic frame for subsequent fabrication. e) Paste the PET substrate cut by the laser cutting machine onto the VHB film. f) Paste the PET reinforced frames on the other side, and apply the carbon grease electrode. After removal, the unilateral actuator is completed. g) Make two unilateral actuators, assemble them to each other oppositely, then get bilateral actuators. h) The bilateral actuators are connected end to end to form a six-teeth robot.


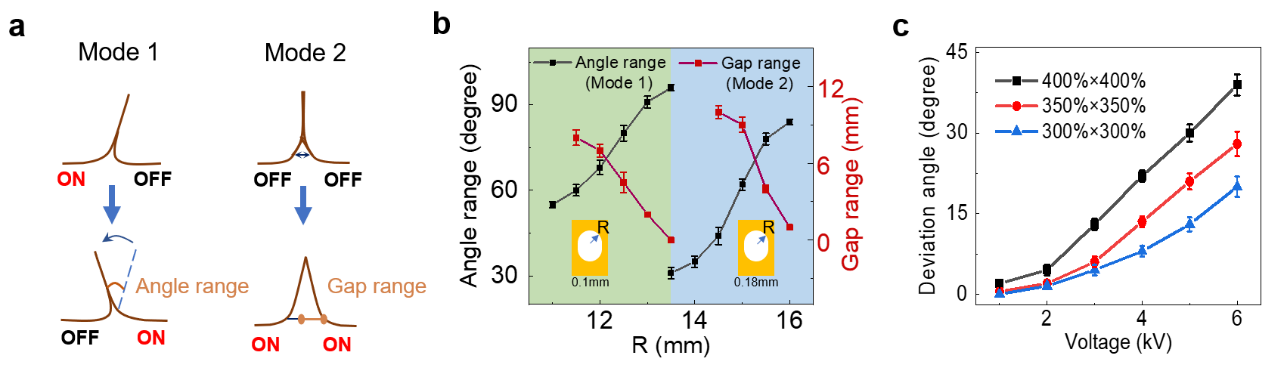


**Fig. S2.** The influence of PET dimensions and dielectric elastomer pre-stretching ratio on the performance of bilateral actuators. a) Schematic diagram of angle range and gap range. b) Gap range is defined as the range of changes in gap distance. For two different thicknesses of PET substrates, using different radii parameters will result in different actuator performance. Considering the bilateral actuators in both modes, the optimal parameters are a radius of 12.5 mm for 0.1 mm thick PET substrate and 15 mm for 0.18 mm thick PET substrate. c) Different pre-stretching ratios can affect the strain of the dielectric elastomer, thereby affecting the deviation angle of the bilateral actuator.


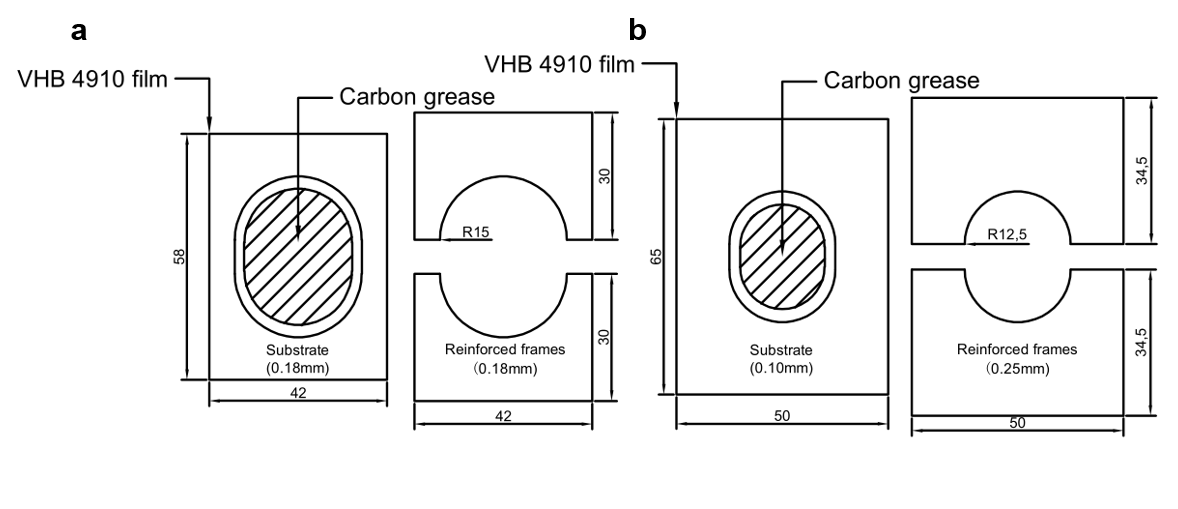


**Fig. S3.** Planar structure design examples of the unilateral actuator for PET substrate with different thicknesses. Different sizes need to be designed according to different thickness of PET substrate. Here are two examples of sizes: a) 0.18mm thick PET substrate and b) 0.10mm thick PET substrate.


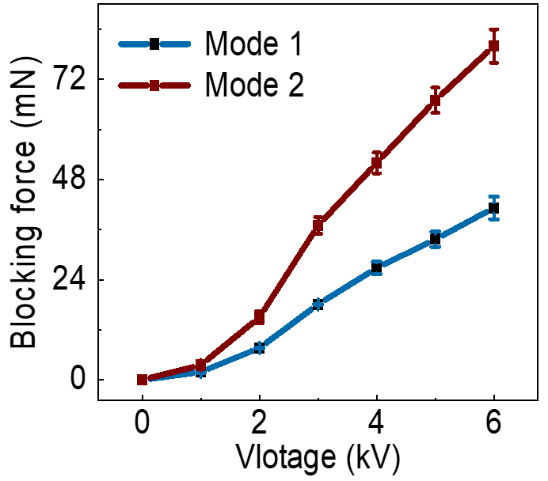


**Fig. S4.** The blocking actuation force under different voltages in two actuation modes. Blocking force refers to the measured force that prevents deformation of the actuator.

**
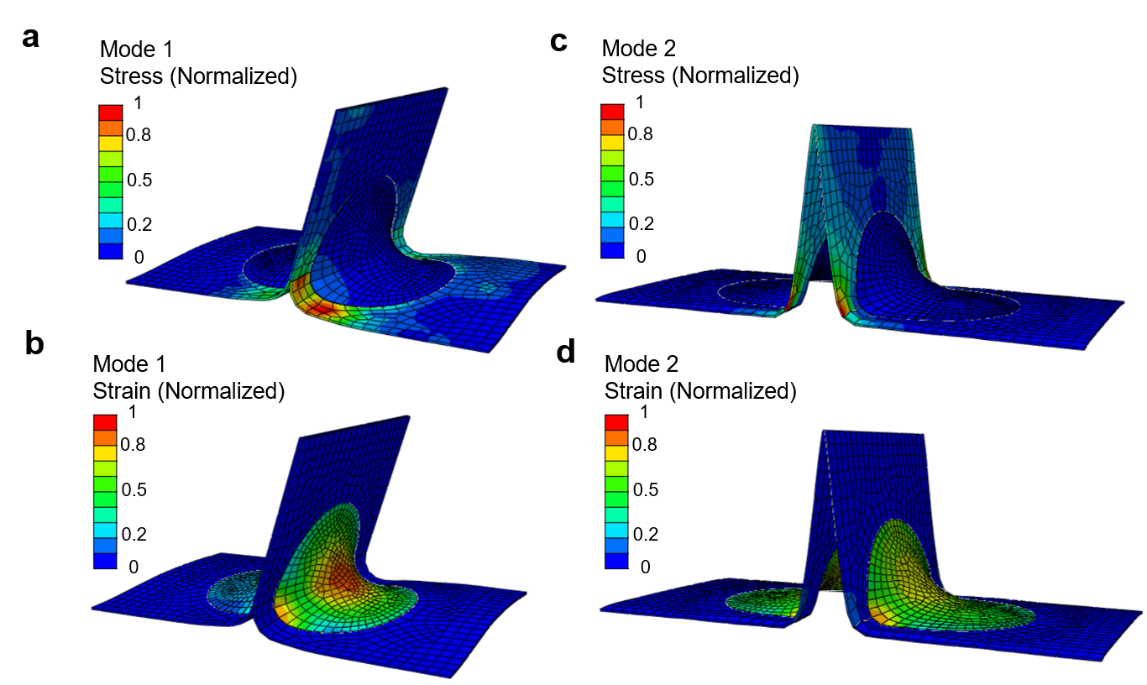
**

**Fig. S5.** Finite element analysis and simulation results of bilateral actuators obtained using ABAQUS. a) The stress distribution of the bilateral actuator working in mode 1. b) The strain distribution of the bilateral actuator working in mode 1. c) The stress distribution of the bilateral actuator working in mode 2. d) The strain distribution of the bilateral actuator working in mode 2.


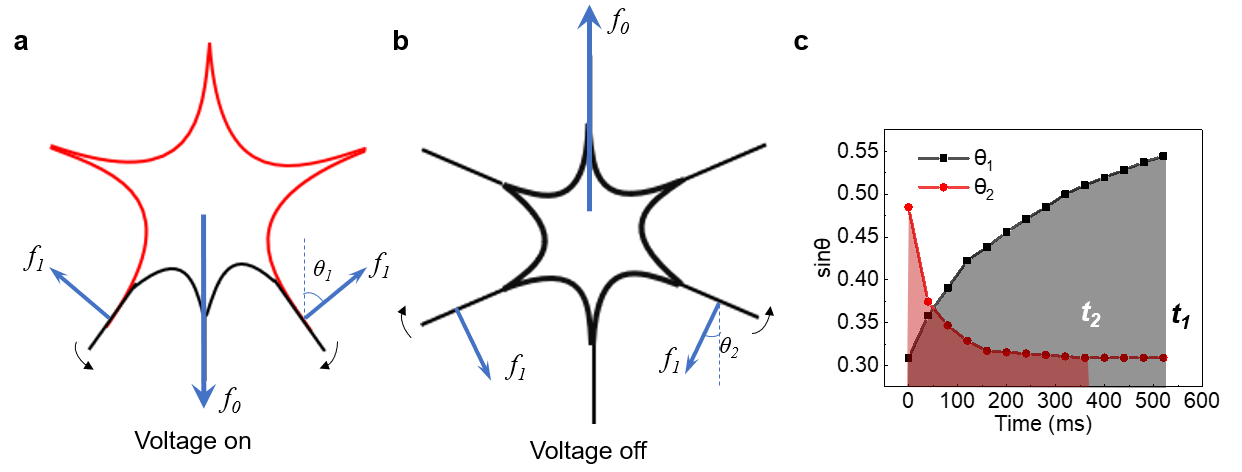


**Fig. S6.** Analysis of the crawling principle of crawling soft robots. a) After applying voltage, the robot is subjected to two types of frictional forces: backward frictional force *f_0_* and frictional force *f_1_* perpendicular to the bilateral actuators. Let the angle between *f_1_* and the forward direction be *θ_1_*. If *f_1_* exists for a duration of *t_1_*, the distance the robot advances during the voltage application process is $d_{1}=\int_{0}^{t_{1}} \frac{\int_{0}^{t_{1}} 2f_{1}sin\theta_{1}dt-f_{0}t}{m}dt+\frac{{(\int_{0}^{t_{1}} 2f_{1}sin\theta_{1}dt-f_{0}t_{1})}^{2}}{2{mf}_{0}}$. b) The process of removing voltage is similar. Let the angle between *f_1_* and the forward direction be *θ_2_*. If *f_1_* exists for a duration of *t_2_*, the distance the robot advances during the voltage removal process is $d_{2}=\int_{0}^{t_{2}} \frac{\int_{0}^{t_{2}} 2f_{1}sin\theta_{2}dt-f_{0}t}{m}dt+\frac{({\int_{0}^{t_{2}} 2f_{1}sin\theta_{2}dt-f_{0}t_{2})}^{2}}{2mf_{0}}$. c) By measuring the changes in various parameters through experiments, it can be found that $\int_{0}^{t_{1}} sin\theta_{1}dt>\int_{0}^{t_{2}} sin\theta_{2}dt$. So, it can be determined that the robot can generate a certain step distance within one actuation cycle.


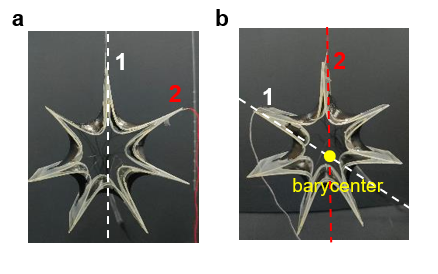


**Fig. S7.** This is a simple method to measure the position of the barycenter. a) First, fix a white flexible cord at one end of the robot (named end 1), and then make the robot hang freely and obtain its optical image. The cord will pass through its barycenter. b) Similarly, use another red flexible cord to fix it at the other end (named end 2) to complete the same operation. Then the position of the barycenter of the robot can be obtained by measuring the intersection of the two lines.


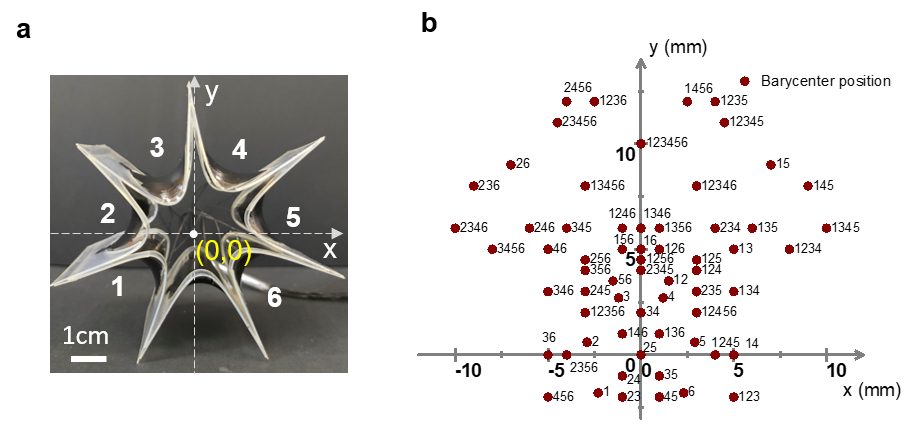


**Fig. S8.** The position of the robot's barycenter in different actuation states. According to this experiment, the most favorable actuating mode for rolling can be designed. a) We number the actuators of the gear-shaped robot clockwise, and select a coordinate system whose origin is the barycenter of the robot in the original state. b) Each point in the figure represents the barycenter position of the robot under different actuation states. For example, "123" means that actuators 1, 2 and 3 are actuated.


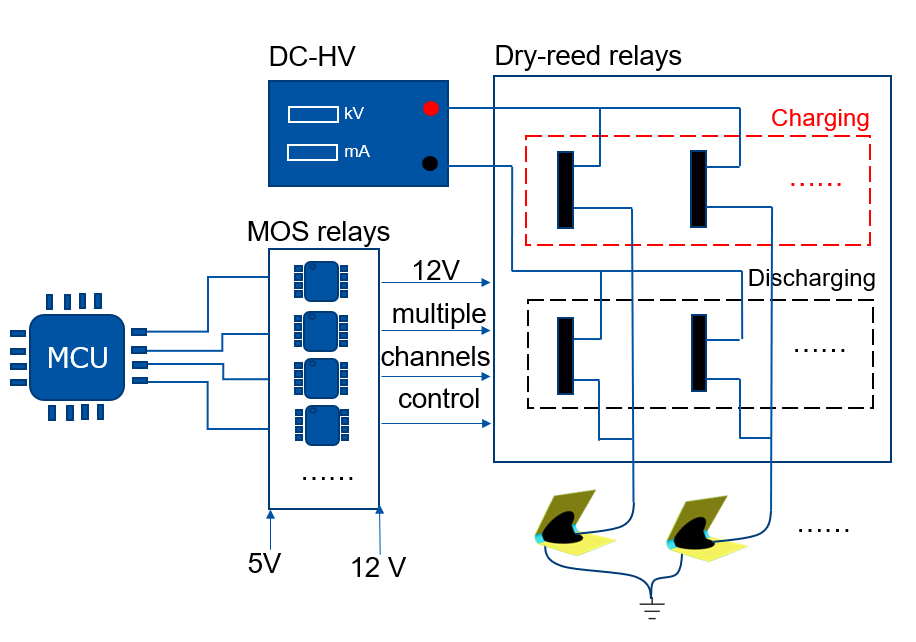


**Fig. S9**. This figure shows the structure diagram of a high voltage multi-channel control circuit. In this figure, DC-HV represents Direct Current High Voltage, and MCU represents Microcontroller Unit. We use MOS relays and dry-reed relays to form a control circuit to multiplex the input DC high-voltage power supply and output multi-channel controllable high-voltage. The ON/OFF of MOS relay is controlled by MCU. Each MOS relay controls a dry-reed relay. The relay is divided into two groups, one for charging process control and the other for discharging process control. An actuator requires a charge relay and a discharge relay for control. This circuit can be expanded into a control circuit with more channels.

**2. Supplementary Tables Description**

**Table S1** Comparison of the DE-based soft robots in controllability.

| Reference | DEA materials | Crawling direction | Turning speed  (°/ s) | Rolling direction | Maximum climbing slope (°) | crawling speed (mm/s) | Step distance (mm) |
| --- | --- | --- | --- | --- | --- | --- | --- |
| [1] | VHB 4910 | Unidirectional | / | / | / | 30 | 15 |
| [2] | VHB 4910 | Unidirectional | 16.19 | / | / | 53 | 2.8 |
| [3] | PDMS | Unidirectional | 9 | / | / | 18 | 0.12 |
| [4] | VHB 4910 | Unidirectional | 62.79 | / | / | 88 | 11 |
| [5] | VHB 4910 | Bidirectional | 1.10 | / | / | 2.5 | 2 |
| [6] | VHB 4910 | / | / | Unidirectional | / | / | / |
| [7] | PDMS | Unidirectional | / | Unidirectional | / | 18.5 | 2.5 |
| This work | **VHB 4910** | **Omnidirectional**  **(360°)** | **>180** | **Bidirectional** | **2** | **4.3** | **6** |

**3. Supplementary Video Description**

**Movie S1.** Angle range comparison of unilateral actuator and bilateral actuator under 6kV voltage of different frequencies.

**Movie S2.** Cycle test of the bilateral actuators in two modes with actuation voltage of 5.5 kV and cycle frequency of 1 Hz.

**Movie S3.** Crawling speed of soft robot under different actuation frequencies.

**Movie S4.** The crawling robot moves in different directions.

**Movie S5.** The bidirectional rolling of the rolling soft robot.

**Movie S6.** Continuous rolling of the rolling soft robot.

**Movie S7.** Rolling behavior of the soft robot on different slope.

**Movie S8.** The transportation process of 40mm-diameter table tennis balls with different weights by the mouthlike robot with different sandpaper inner walls.

**References**

1. Nguyen, C. T., Phung, H., Nguyen, T. D., Jung, H., & Choi, H. R. Multiple-degrees-of-freedom dielectric elastomer actuators for soft printable hexapod robot. *Sensors and Actuators A: Physical* **267**, 505-516 (2017).
2. Hu, T., Lu, X., & Liu, J. Inchworm‐Like Soft Robot with Multimodal Locomotion Using an Acrylic Stick‐Constrained Dielectric Elastomer Actuator. *Advanced Intelligent Systems* **5**, 2200209 (2023).
3. Ji, X. et al. An autonomous untethered fast soft robotic insect driven by low-voltage dielectric elastomer actuators. *Science Robotics* **4**, eaaz6451 (2019).
4. Gu, G., Zou, J., Zhao, R., Zhao, X., & Zhu, X. Soft wall-climbing robots. *Science Robotics* **3**, eaat2874 (2018).
5. Guo, Y., Guo, J., Liu, L., Liu, Y., & Leng, J. Bioinspired multimodal soft robot driven by a single dielectric elastomer actuator and two flexible electroadhesive feet. *Extreme Mechanics Letters* **53**, 101720 (2022).
6. Li, W. B., Zhang, W. M., Zou, H. X., Peng, Z. K., & Meng, G. A fast rolling soft robot driven by dielectric elastomer. *IEEE/ASME Transactions on Mechatronics* **23**, 1630-1640 (2018).
7. Duduta, M., Berlinger, F., Nagpal, R., Clarke, D. R., Wood, R. J., & Temel, F. Z. Tunable multi-modal locomotion in soft dielectric elastomer robots. *IEEE Robotics and Automation Letters* **5**, 3868-3875 (2020).
